# Automatically detecting bregma and lambda points in rodent skull anatomy images

**DOI:** 10.1371/journal.pone.0244378

**Published:** 2020-12-29

**Authors:** Peng Zhou, Zheng Liu, Hemmings Wu, Yuli Wang, Yong Lei, Shiva Abbaszadeh

**Affiliations:** 1 Department of Electrical and Computer Engineering, University of California, Santa Cruz, Santa Cruz, California, United States of America; 2 Department of Nuclear, Plasma, and Radiological Engineering, University of Illinois at Urbana-Champaign, Urbana, Illinois, United States of America; 3 Department of Neurosurgery, Stanford University, Palo Alto, California, United States of America; 4 State Key Laboratory of Fluid Power and Mechatronic Systems, School of Mechanical Engineering, Zhejiang University, Hangzhou, China; University of Engineering & Technology, Taxila, PAKISTAN

## Abstract

Currently, injection sites of probes, cannula, and optic fibers in stereotactic neurosurgery are typically located manually. This step involves location estimations based on human experiences and thus introduces errors. In order to reduce localization error and improve repeatability of experiments and treatments, we investigate an automated method to locate injection sites. This paper proposes a localization framework, which integrates a region-based convolutional network and a fully convolutional network, to locate specific anatomical points on skulls of rodents. Experiment results show that the proposed localization framework is capable of identifying and locatin bregma and lambda in rodent skull anatomy images with mean errors less than 300 *μm*. This method is robust to different lighting conditions and mouse orientations, and has the potential to simplify the procedure of locating injection sites.

## Introduction

Stereotactic neurosurgery is a surgical intervention technique with minimal invasion that uses three-dimensional coordinate systems to apply treatments on small targets inside the skull. It is widely used in both preclinical and clinical studies. In preclinical animal studies, researchers use stereotactic neurosurgery to introduce fluids to the brain and stimulate specific brain sites. As an example of clinical applications, stereotactic neurosurgery is used to treat Parkinson’s disease in procedures such as Pallidotomy, in which doctors introduce a small electrical probe in patients’ globus pallidus and apply treatments by heating the probe and destroying brain cells around it [1].

**Fig 1:**
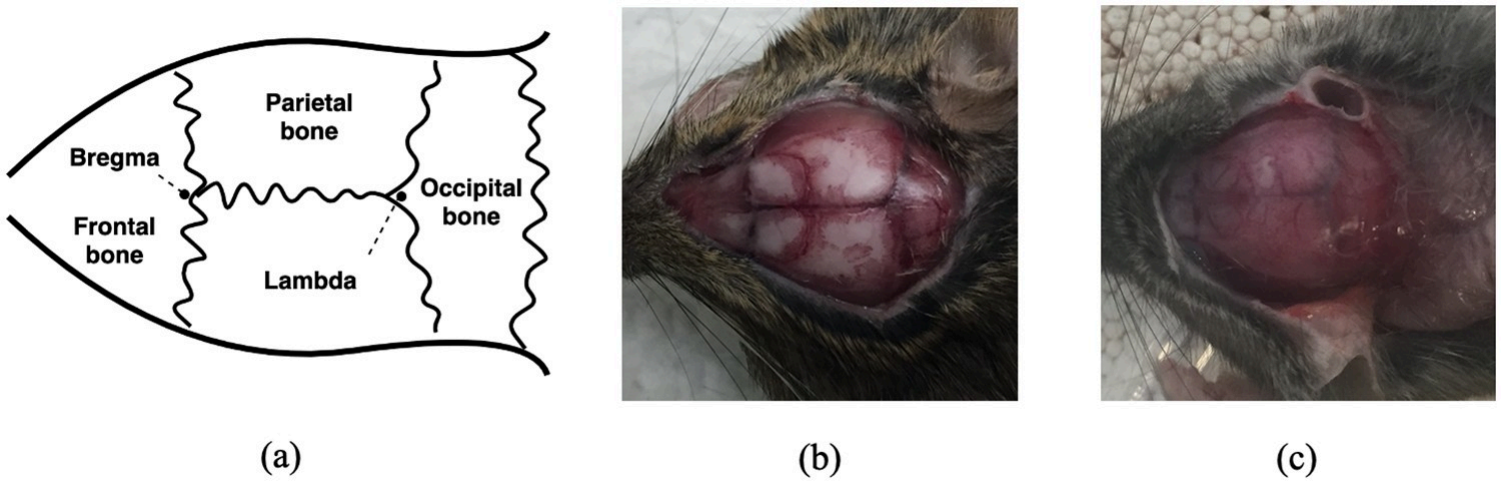
(a) Schematic of bregma and lambda. (b) A subject with a clear view of bregma and lambda. (c) A subject with a obscured view of bregma and lambda.

In the stereotactic neurosurgical procedure, a critical step is the insertion of surgical instruments such as probes and optical fibers. This step is associated with human visual estimation and depends on investigator experience, which introduces positioning errors for the injection site. For example, the stereotactic procedure in rodents often involves inserting probes into the brain relative to the position of bregma and lambda, which are two specific anatomical points on the skull. Although these two points are theoretically easy to find (Fig 1a), individual anatomical variations between subjects make these two points difficult to locate in real applications (Fig 1b and 1c). Previous work has investigated computer-guided stereotactic positioning of injection sites based on skull model registration [2–5] and template matching [6]. With a carefully defined field of view, previous methods [6] can identify the suture patterns of the skull and calculate the position of the injections. However, this method [6] is vulnerable to rotating and shifting of the template. The orientation and angle need to be carefully aligned between the template and the experimental image. Recent developments of needle insertion and deep learning provide an alternative way to insert surgical instruments automatically. The problem can be separated into two parts, (i) determining the insertion location, and (ii) moving and manipulating the instrument to the location site. The former task can be accomplished by taking images of the subject with cameras, and passing those images through deep learning algorithms. The latter task, which includes the needle insertion procedure, can be conducted by a manipulator with multiple degrees of freedom [7].

Image segmentation has been used to identify and locate features in images [8, 9]. Since 2012, deep convolutional networks have shown great advantages in image recognition tasks [10–13] and caught up with humans in classification performance [14]. In image recognition, deep networks are designed to classify images into different categories. By modifying the structures of these deep classification networks, their application scenarios can be expanded into object detection and image segmentation. In object detection, deep networks not only predict the category label of the image, but also draw bounding boxes in the image to locate the object. A series of network structures have been developed for object detection [15–18]. Their accuracies were improved over the past years, and their efficiencies were improved to achieve real-time implementation. In contrast to object detection, image segmentation networks identify image features in a different manner: they predict a category label for each pixel in the image [8]. Generally, image segmentation networks use a series of convolution layers to extract features from an image, and then use a series of deconvolution layers to reconstruct a segmentation of the original image with each pixel being a category label.

In this work, a localization framework is proposed to calculate the coordinates of bregma and lambda in rodent images. This localization framework was composed of two stages: in stage one, a region-based convolutional network (faster-rcnn [17]) was used to detect the skull region in images; in stage two, a fully convolutional network (FCN) was modified from the implementation described by Long, Shelhamer, and Darrell [8] to segment bregma and lambda in the skull region. Faster-rcnn is a widely used object detection algorithm, which could achieve faster training speed and better performance compared to other object detection neural networks. We leverage these advances of faster-rcnn and apply them to our task to find only the skull region of interest (ROI) of each image. For stage two, we chose the FCN as it is one of the most important architectures in image segmentation. Compared with the original FCN implementation, residual networks [13], bottleneck design [13], and batch normalization [19] are employed in this work to increase training performance. With this two-stage framework rather than an end-to-end approach (applying the original image to the FCN), we can obtain higher accuracy, and save computing resources and training time.

## Materials and methods

### Ethics statement

No live animal was used for this study. All images were acquired from sacrificed animals previously approved for other research protocols by the Stanford University Administrative Panel on Laboratory Animal Care [20].

### Dataset

In this paper, 93 rodent images were collected for training and testing the localization framework. Those images were from mice (male and female, age 8-28 weeks, various strains) that were previously sacrificed within 2 days for other experiment purposes. The raw images had dimensions of 2448 × 3264 × 3 (length × width × RGB color). The images were acquired with the camera from a hand-held iPhone 6 (Apple Inc.). The images collected by smartphones are proved to be useful with neural networks for the suitable medical applications [21, 22]. The light conditions and mouse orientations are not controlled as we would like our model to be flexible and robust.

**Fig 2:**
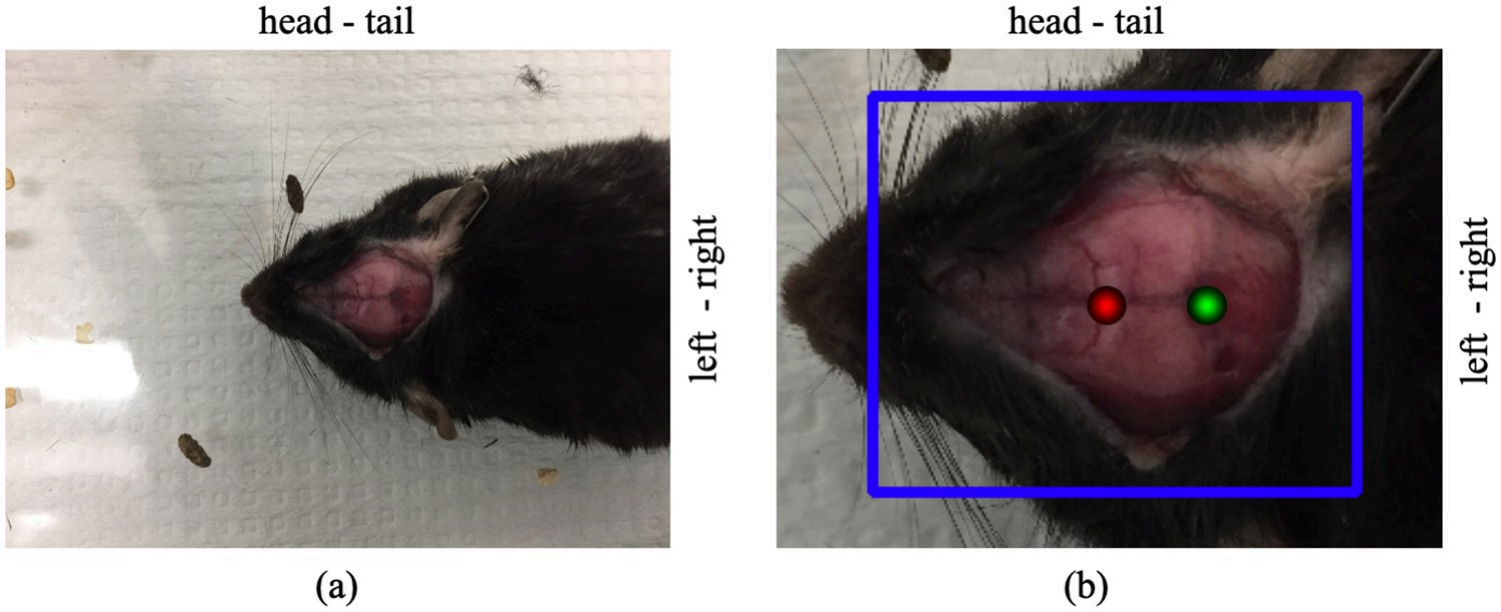
(a) A rodent image example. (b) The skull region of the rodent image with bounding box (the blue rectangle) and bregma/lambda masks (the red/green circles) manually labeled. In the red and green circles, the closer a pixel is to bregma/lambda point, the larger the value (i.e., brighter) is assigned to the labeled pixel. Therefore, the edges of the two circles are dark while the center is bright. The bounding box is used to train the faster-rcnn in stage one, and bregma/lambda masks are used to train the FCN in stage two.

For each image, a bounding box is labeled to denote the skull area in stage one. In stage two, the label represents a two-dimensional Gaussian distribution whose mean is denoted as bregma/lambda points and has a standard deviation of 20 pixels (Fig 2). A binary label was previously examined for a classification task [23]. It was shown that FCN would output pixel-wise segmentations with highest probability scores to the bregma/lambda areas and it can assign moderate probability score to areas similar to bregma or lambda. In some test cases this led to the production of more than two corresponding masks [23]. With gaussian labels, we transfer the classification task to a regression task, which could embrace more useful information during the training and ultimately contribute a better performance of our network. The coordinates of bregma and lambda are provided by an expert—a trained neurosurgeon—in the full-resolution images.

### Localization framework

**Fig 3:**
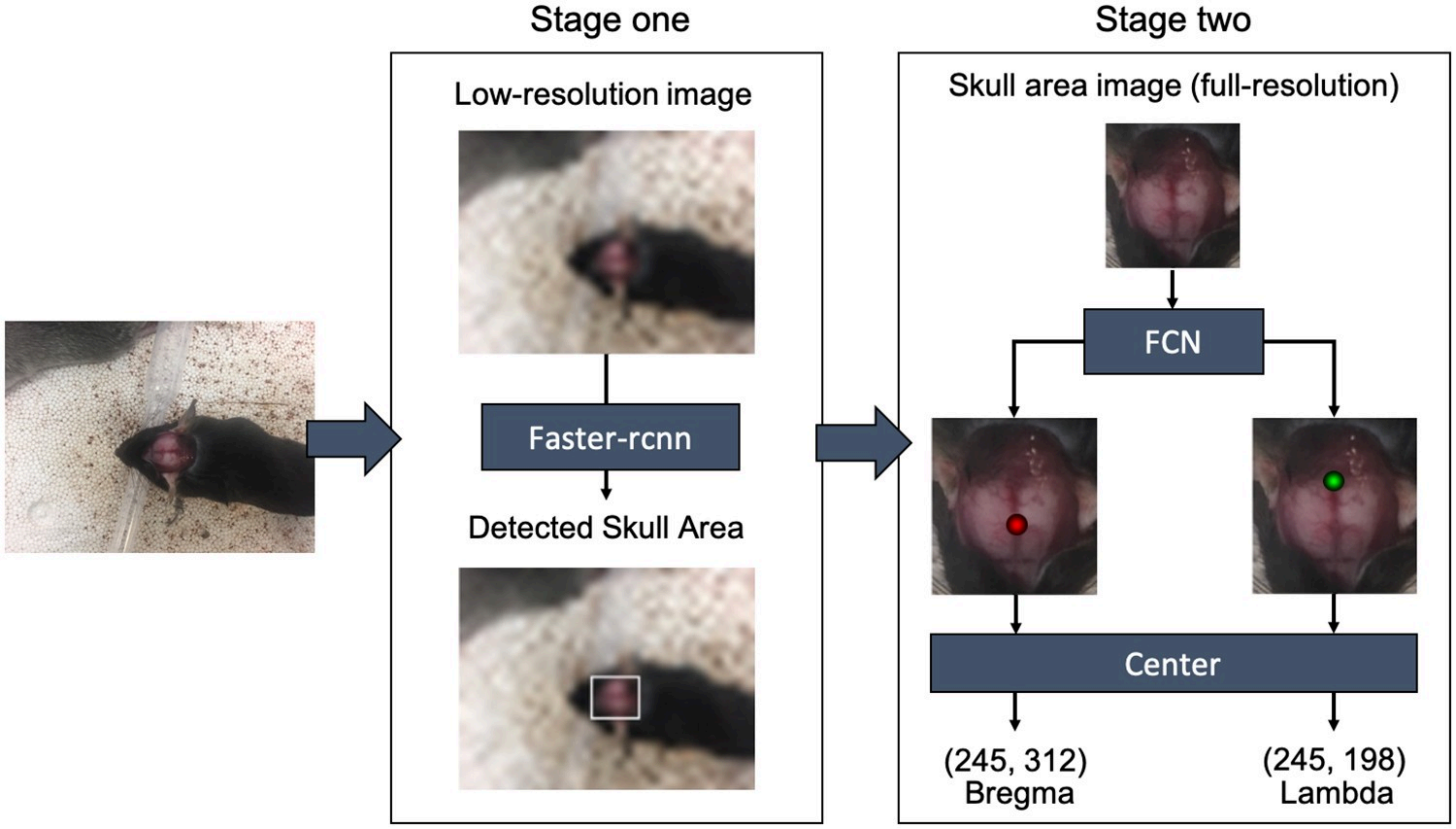
The two-stage localization framework. In stage one, the localization framework detects the skull area in the low-resolution image using faster-rcnn. In stage two, the localization framework segments bregma and lambda from the full-resolution skull area image using FCN, and the coordinates of bregma and lambda are determined as the maximum value of the remaining segmented area.

Although the FCN can be directly used to locate bregma and lambda in raw input images, it is not efficient enough because the skull of the mouse only takes up a small portion of the area in the raw input images (Fig 2). To speed up the locating process, we applied a two-stage framework that integrates a faster-rcnn and a FCN together to locate bregma and lambda in mouse images as shown in Fig 3.

In stage one, a low-resolution version of the input image was generated with reduced image size, and a faster-rcnn was applied to locate the skull area in the low-resolution image. The identified skull area’s coordinates in the low-resolution image were then transformed back to the full-resolution image to crop the skull area from the full-resolution image.

**Fig 4:**
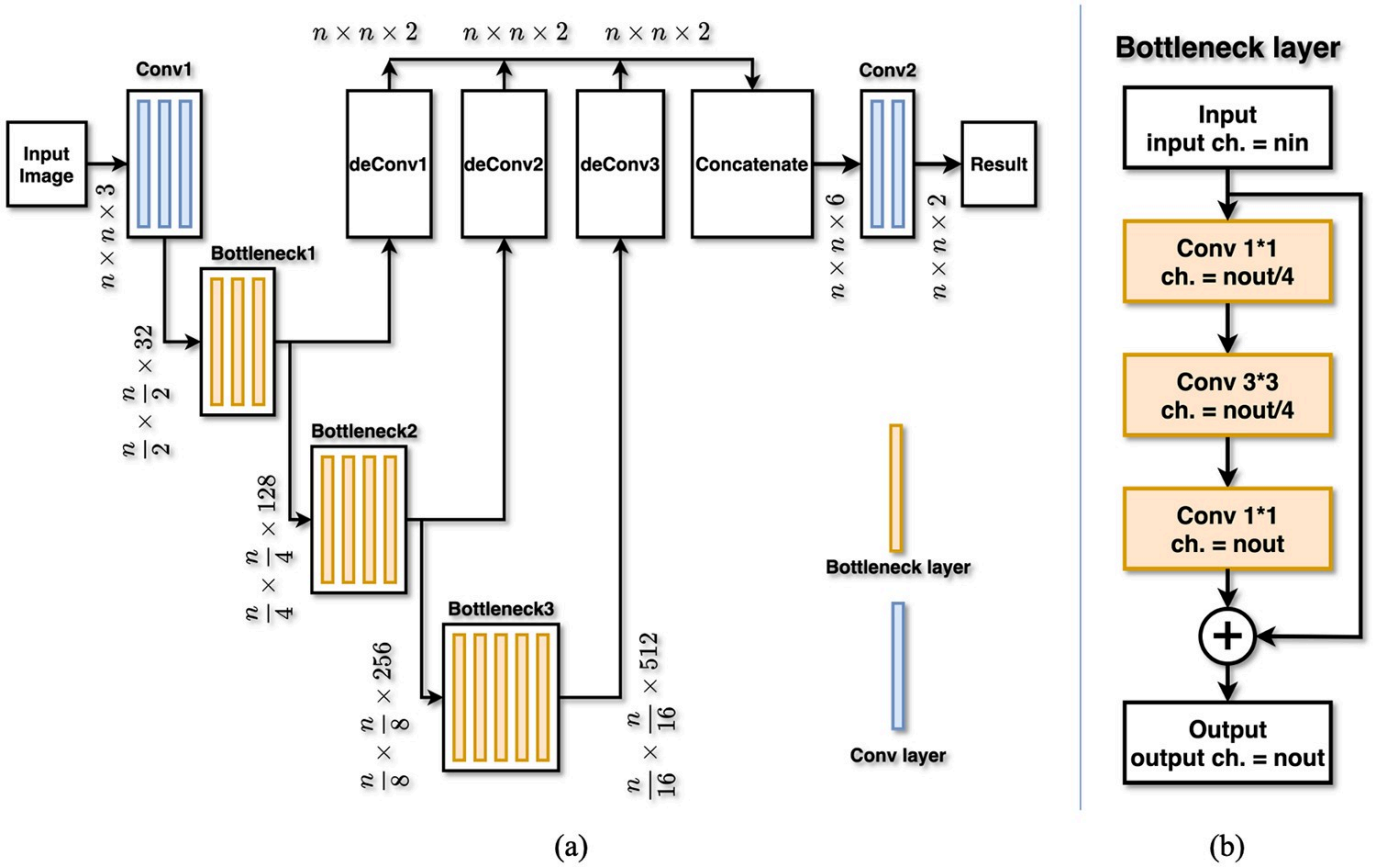
The fully convolutional network. (a) Detailed structure of the FCN used in stage two of the localization framework. Blue slices represent convolution layers, and orange slices represent bottleneck layers. (b) Detailed structure of one bottleneck layer. Input and output channel numbers are *nin* and *nout*, respectively.

In stage two, the cropped skull area from the full-resolution image was fed into a FCN for pixel-wise segmentation of bregma and lambda. The structure of the FCN adopted in this work is illustrated in Fig 4. Input images (dimension: *n* × *n* × 3) were first processed by three 3 × 3 convolution layers in the Conv-1 block to reduce the image size by an order of 2 and to increase the channel number from 3 to 32. Then the data were processed by the Bottleneck-1 block which contained three bottleneck layers to reduce the image size by an order of 2 and to increase the channel number from 32 to 128. After the Bottleneck-1 block, the data were successively processed by Bottleneck-2 (4 bottleneck layers) and Bottleneck-3 (6 bottleneck layers) blocks. In each of the two Bottleneck blocks, the image size was reduced and the channel number was increased by an order of 2. The output of the three Bottleneck blocks (Bottleneck-1, Bottleneck-2, Bottleneck-3) was processed by 3 de-Convolution blocks to restore the original input image size with 2 channels. These three outputs were concatenated together and processed by another two convolution layers in the Conv-2 block to generate the final result. This final result had the same size (*n* × *n*) in each channel as the input image and contains two channels. These two channels represented the probability of a pixel belonging to bregma and lambda. The final coordinates of bregma and lambda were determined as the maximum value of the predicted region. All the convolution/deconvolution layers were followed by batch normalization layers and rectified activation layers except the last convolution layer. There is no activation layer after the last convolution layer, which fits the label based on a Gaussian distribution. We applied residual networks [13], bottleneck design [13], and batch normalization [19] to FCN to improve training performance.

### Implementation

For implementation of this framework, in stage one, 93 images were separated into training (60 images) and testing (33 images). The results show that the skull area of all images is clearly detected. Original training images with size of 2448 × 3264 × 3 were first resized to 612 × 816 × 3 and fed into the faster-rcnn to train the model from scratch. The implementation of faster-rcnn followed the approach described by Ren, He, and Girshick [17]. The faster-rcnn output the rectangle coordinates of the skull area in low-resolution rodent images. These coordinates were then transformed back to the full-resolution image and the area was extended to generate a 640 × 640 × 3 cropping of the original image based on the center of the rectangle. This cropping was further down-sampled to 256 × 256 × 3 to generate the input image for stage two. In the training process of the FCN, 93 images were separated into training (80 images) and testing (13 images) subsets. To provide learning robustness [24], each training image was augmented 100 times with randomly flipping/rotating/shifting the cropping of the original images which yields 8000 images. The 8000 images dataset was separated into 6000 augmented images for the training subset and 20 original images for the validation subset for 4-fold cross validation, as shown in Table 1. The remaining 13 images are only for testing and evaluating the network performance. Mean square error was used as the loss function of the FCN to be compatible with the regression task, and Adam optimizer with default learning rate (0.001) was applied to train the FCN. The output of the FCN has dimensions of 256 × 256 × 2 with the first and the second channels representing the probability of being bregma and lambda. We add the third channel as all zero to save as an image. This 256 × 256 × 3 image was then resized back to its original size, 640 × 640 × 3, and the pixel with maximum value in each channel is determined as the coordinate of bregma and lambda. The faster-rcnn was implemented using Keras/tensorflow package, and the FCN was implemented using tensorflow package. The training was based on a system with Windows 10 and NVIDIA GeForce RTX 2070. It took about 10 hours to train 50 epochs and 20 mins to have a stable model in stage one. In stage two, it required about 1 hour to train 50 epochs and 15 mins to have a stable model.

**Table 1:** The composition of training (6000 images), validation (20 images), and test (13 images) datasets.

	Subset 1	Subset 2	Subset 3	Subset 4	Subset 5
Fold 1	Validation	Train	Train	Train	Test
Fold 2	Train	Validation	Train	Train	Test
Fold 3	Train	Train	Validation	Train	Test
Fold 4	Train	Train	Train	Validation	Test

## Results and discussion

### Framework performance of different labels

Table 2 presents the localization errors on testing data for different standard deviations of the Gaussian distribution. When the standard deviation is too small (5 pixels), the training performance has a high level of error. We believe the reason is that the information provided by this case is not enough for the neural network to learn. We chose the standard deviation as 20 pixels because the localization of both bregma and lambda have relatively small mean error.

**Table 2:** Localization errors of different standard deviation labels.

Standard deviation (pixels)	5.00	10.00	20.00	40.00
Bregma mean error (pixels)	404.07	10.55	10.12	11.45
Lambda mean error (pixels)	509.35	14.72	10.15	10.23
Bregma max error (pixels)	494.49	25.96	30.81	30.00
Lambda max error (pixels)	549.66	31.38	22.00	21.10

### Training and validation loss

**Fig 5:**
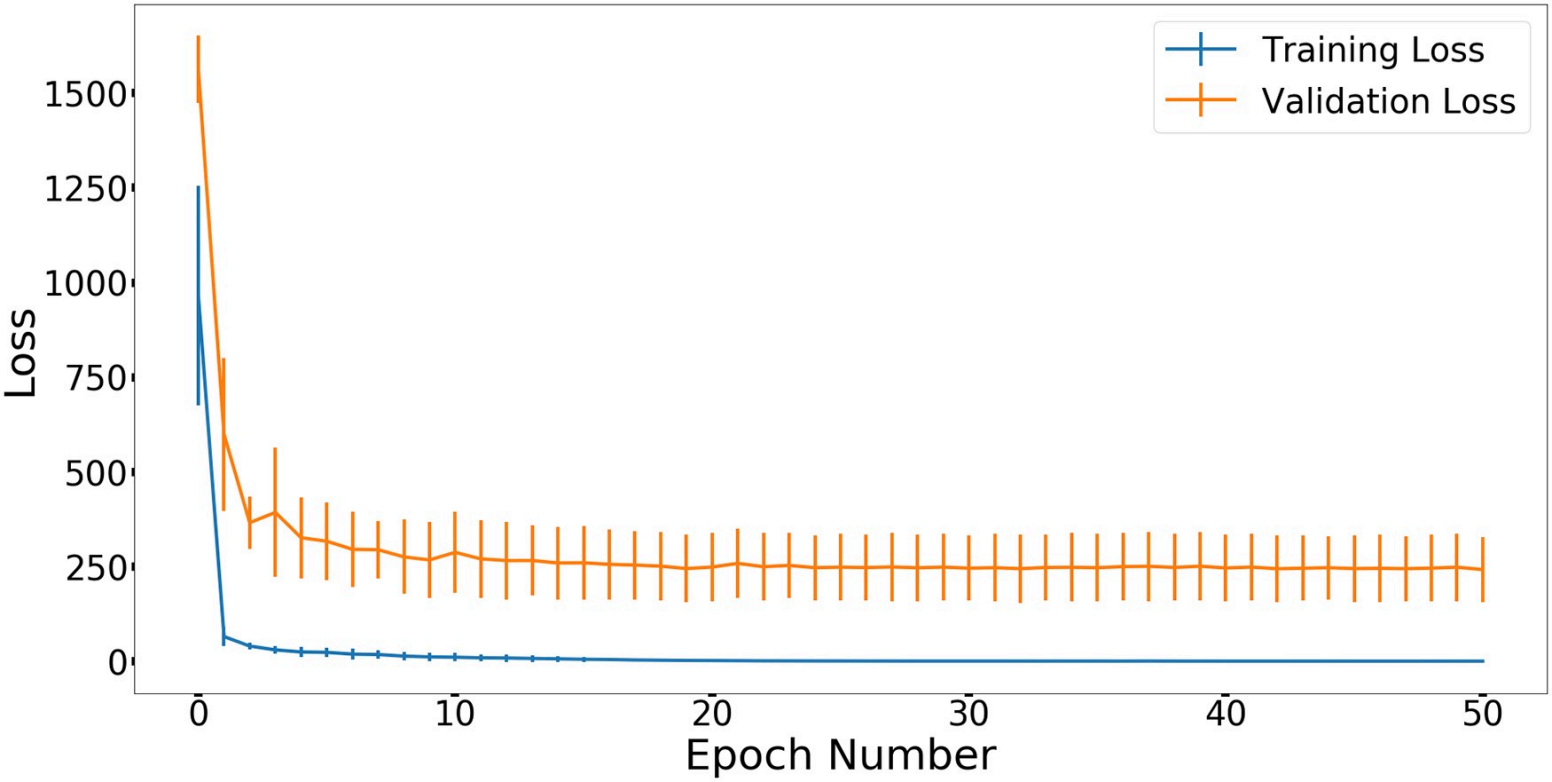
Mean and standard deviation of training loss and validation loss in 50 epochs across the 4-fold cross validation shown as error bars.

Fig 5 shows the mean and standard deviation of training loss and validation loss in stage two during the 50 epochs. After epoch 11, the model is very stable. The training and validation loss decreased as the number of epochs increased.

### Result analysis

**Fig 6:**
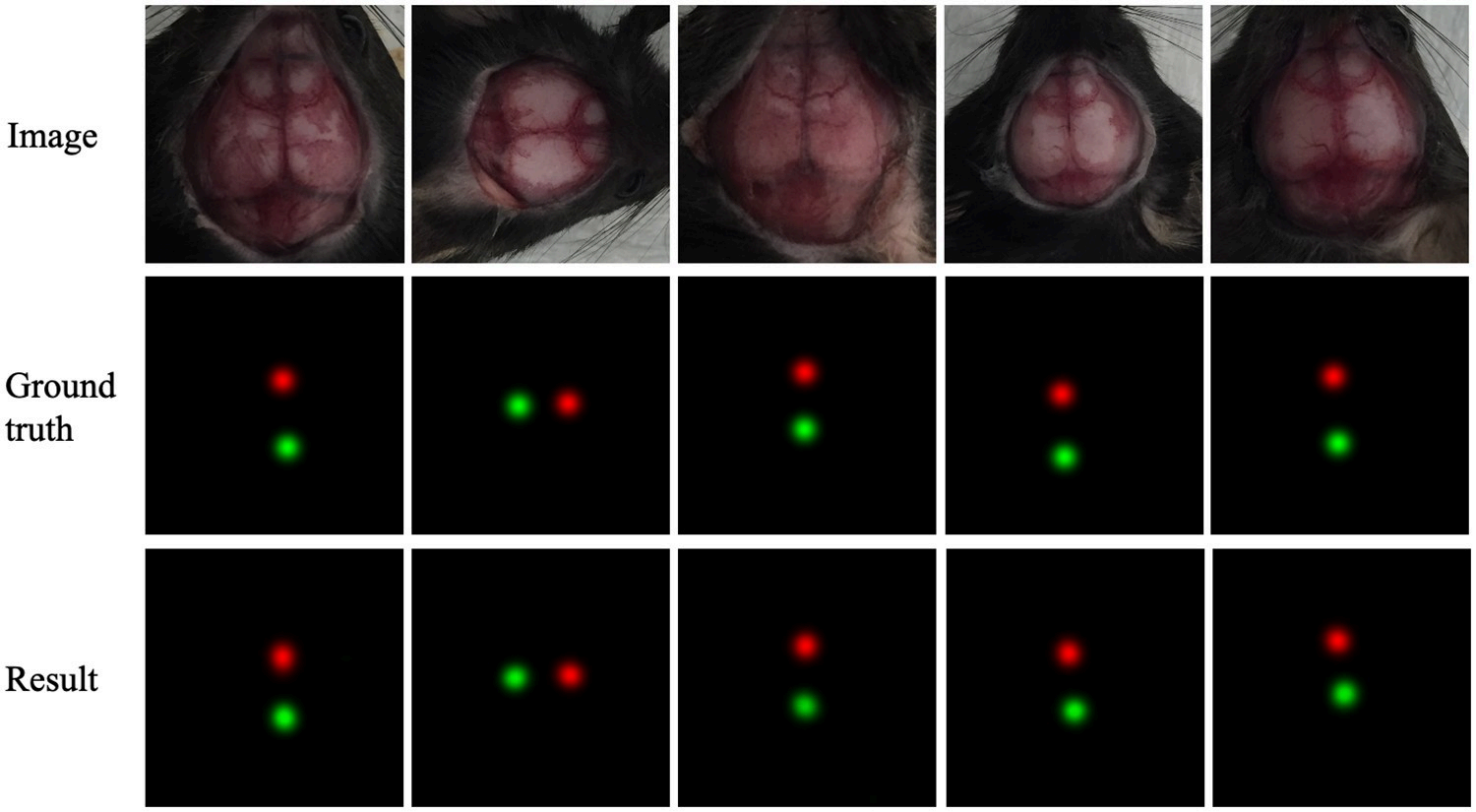
Examples of input image, ground truth, and the result of the stage two.

Fig 6 presents some examples of input image, ground truth, and the result of stage two. The centers of the red and green circles denote bregma and lambda positions, respectively.

**Fig 7:**
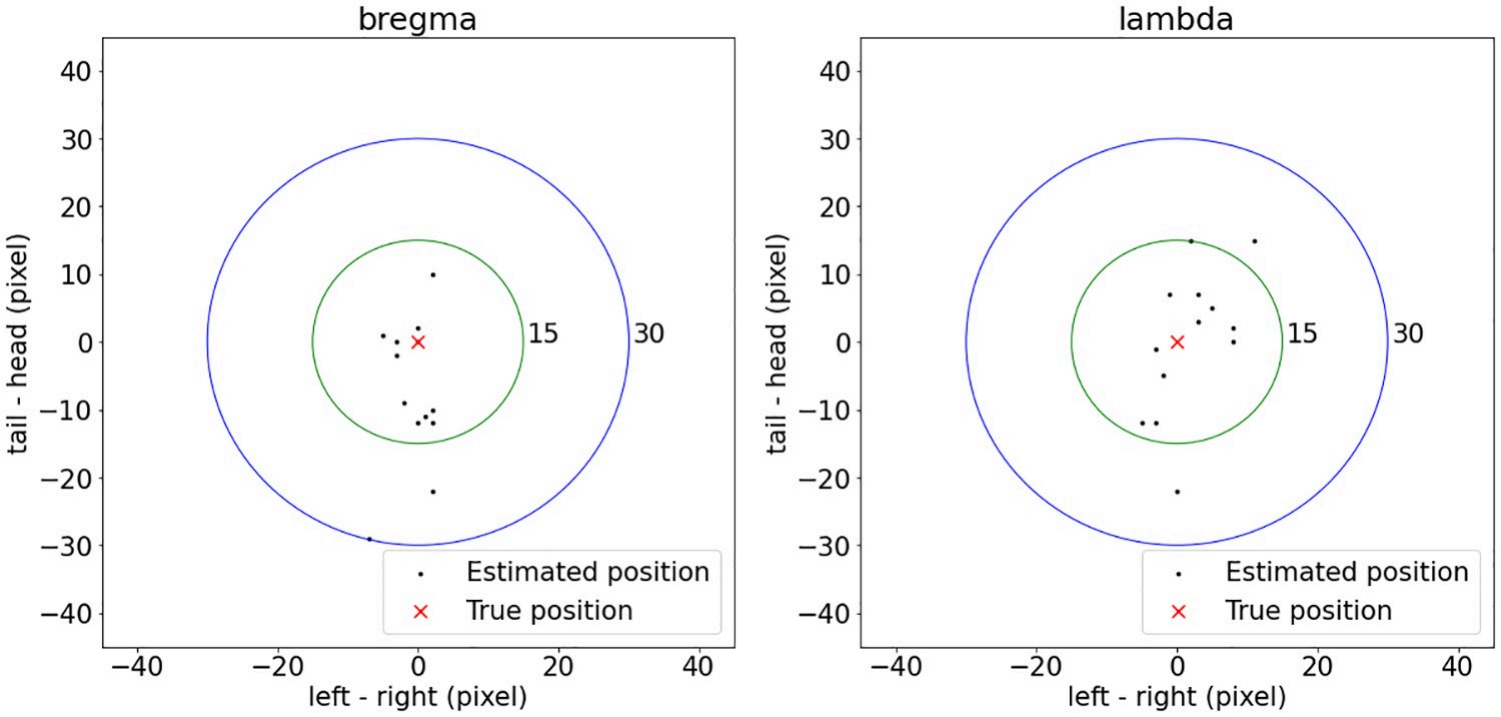
Localization results of the localization framework for 13 testing images. The left panel shows the localization error of bregma, and the right panel shows the localization error of lambda. Every black dot represents a localization result for a rodent image, and the red cross represents the ground truth position of bregma and lambda.

Fig 7 plots the localization error of the localization framework on the testing dataset. Bregma had mean localization error of 10.12 pixels and max localization error of 30.81 pixels, while lambda had mean localization error of 10.15 pixels and max localization error of 22.00 pixels. In this dataset, the mean distance between bregma and lambda was about 4.2 *mm*, which was measured to be about 163.20 pixels in the image. The conversion between pixel and length was 1 pixel per 25.74 *μm*. With this conversion, the localization framework achieved mean localization accuracy of 260.44 *μm* and 261.21 *μm* for bregma and lambda, respectively. bregma and lambda had different localization error behaviors. As shown in Fig 7, errors of bregma mostly came from the tail-head direction, while errors of lambda were more spread along both the tail-head and the left-right directions. A possible explanation was that the sagittal suture was generally clearly visible in images and was a good reference for determining the left-right position for both bregma and lambda. However, the intersection between sagittal suture and coronal suture (this intersection point is defined as bregma) was sometimes not clear in images, and blood vessels along the sagittal suture might also look similar to that intersection. These made it difficult to determine the tail-head coordinate of bregma.

We have included the label results from three students who are studying neurosurgery. The comparison is shown in Table 3. We can find from the Table 3 that although the lambda mean error by student 3 is more accurate than our result, our approach has less mean error for both bregma and lambda than student 1 and 2, also less bregma mean error than student 3. Moreover, we can find that the localization of lambda is not stable by humans, as student 1 and 2 give a large error, but student 3 gives a small error. However, our approach gives a relatively stable performance as the max error is 22.00 pixels, which is better than the results by students.

**Table 3:** The comparison of the localization error between our results and humans.

	Student 1	Student 2	Student 3	Our result
Bregma mean error (pixels)	11.10	11.49	11.56	10.12
Lambda mean error (pixels)	14.18	17.99	8.98	10.15
Bregma max error (pixels)	31.26	24.19	34.21	30.81
Lambda max error (pixels)	30.07	29.07	24.19	22.00

We also implemented an end-to-end approach. We cropped the raw 2448 × 3264 × 3 image to be 2048 × 2048 × 3 and resized it to be 640 × 640 × 3 as the input image to train the FCN, and then resized it back to determine the final coordinates of bregma and lambda. The comparison between these two approaches is shown in Table 4. Compared to the end-to-end neural network, the two-stage method has less training time to obtain a stable model (early stopping time), requires less dedicated GPU memory, and achieves high accuracy. Therefore, it is more suitable to our application.

**Table 4:** The comparison between the two-stage and the end-to-end approaches.

	Two-stage	End-to-end
Training time to obtain stable model (hours)	0.25	1.00
Dedicated GPU memory (GB)	1.9	6.9
Bregma mean error (pixels)	10.12	13.00
Lambda mean error (pixels)	10.15	13.47
Bregma max error (pixels)	30.81	45.00
Lambda max error (pixels)	22.00	43.42

## Conclusion and future work

In this study, a two-stage localization framework was built to estimate bregma and lambda positions in rodent skull anatomy images. This framework utilized faster-rcnn to detect the skull area, FCN to pixel-wisely segment bregma and lambda regions, and chose the pixel with maximum probability as the final coordinates of bregma and lambda. In the experiment, this framework achieved mean localization accuracy of 10.12 pixel (roughly 260.44 *μm*) and 10.15 pixel (roughly 261.21 *μm*) for bregma and lambda respectively. Although the experiment was conducted upon rodent images for detecting bregma and lambda, we believe, with proper training data, this framework is also applicable to detect other anatomical points in preclinical studies. The results also show the potential of the localization framework with low-cost imaging equipment to accurately locate anatomical points in preclinical neurosurgery studies. We believe the proposed automated detection approach can be utilized in a robotic injection system with implementing an application that provides feedback to the micropositioner in a small animal stereotaxic instrument.

The code, raw images, and results of this work are available in a Github repository https://github.com/rillab/SMALL_ANIMAL.

## Supporting information

S1 Raw images (PDF)
