# Supplementary figures and images for: Automatically detecting bregma and lambda points in rodent skull anatomy images
Source: PLoS One. 2020 Dec 29;15(12):e0244378. doi: 10.1371/journal.pone.0244378 (PMC7771702; doi:10.1371/journal.pone.0244378)

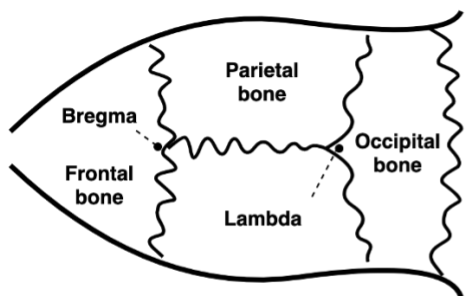

(a)

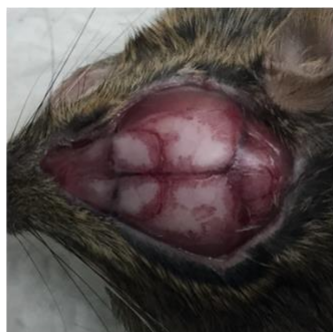

(b)

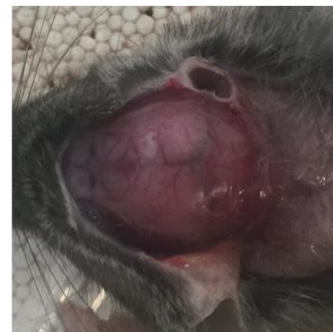

(c)

Fig 1

head - tail

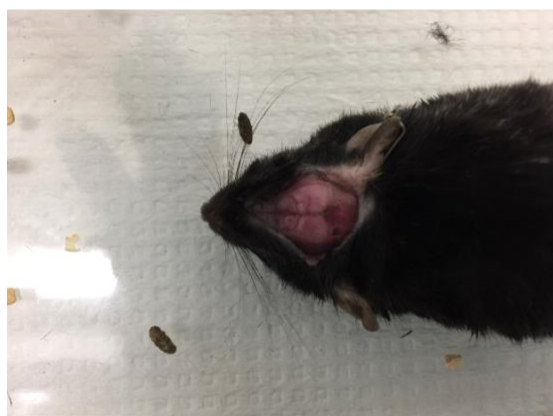

(a)

head - tail

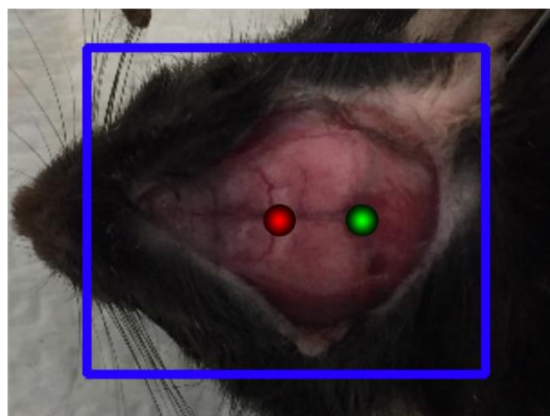

(b)

Fig 2

left - right

left - right

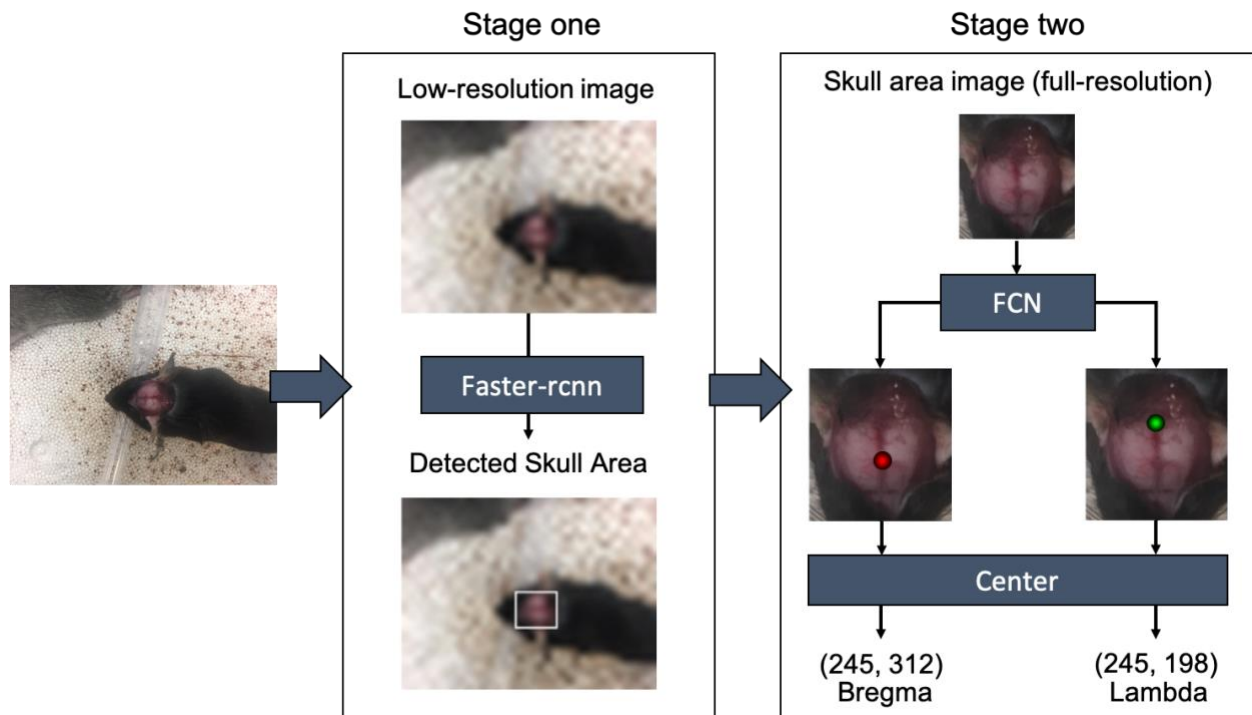

Fig 3

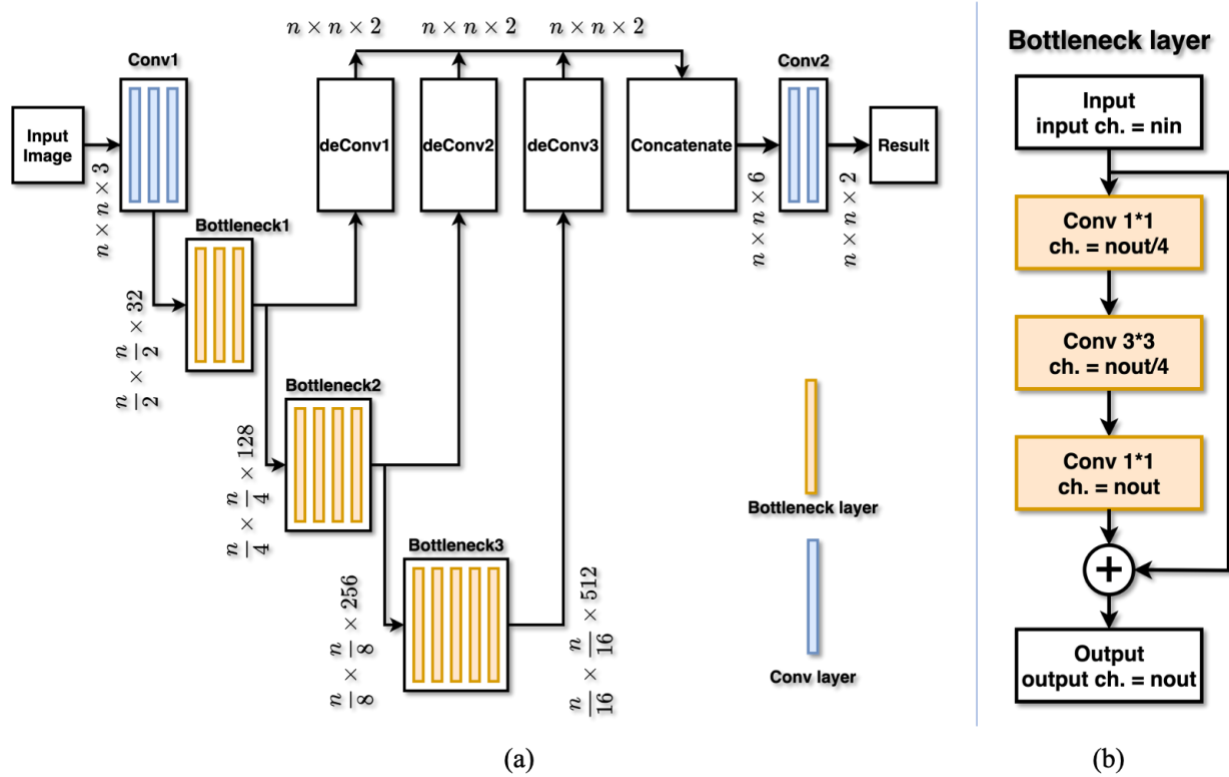

Fig 4

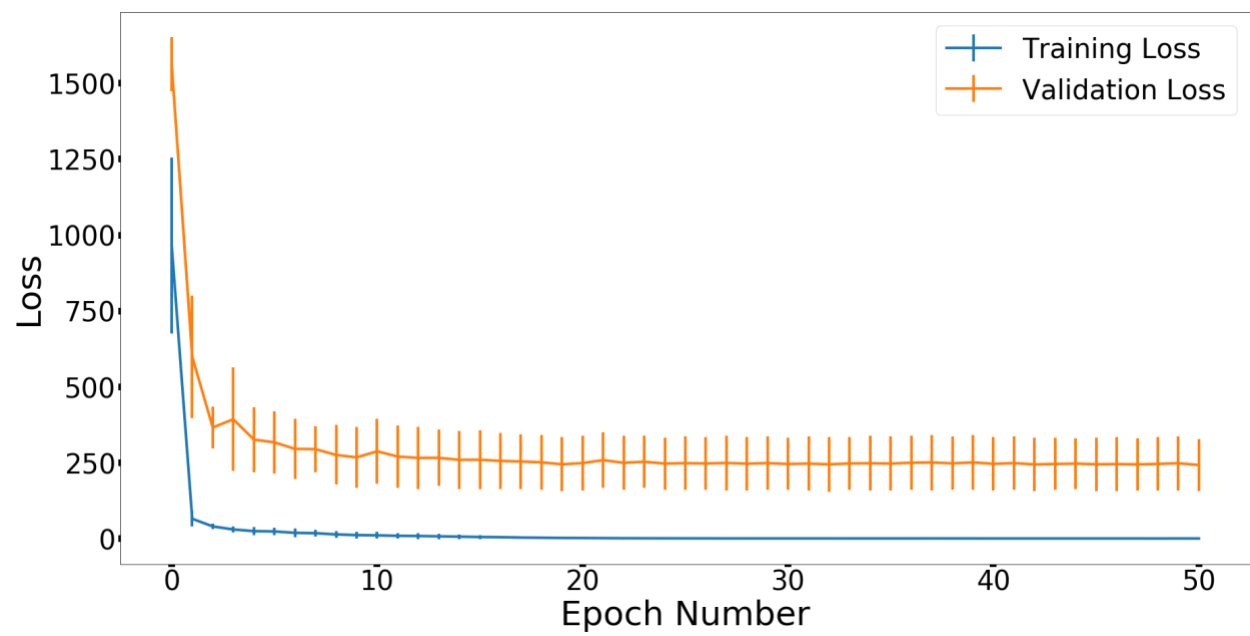

Fig 5

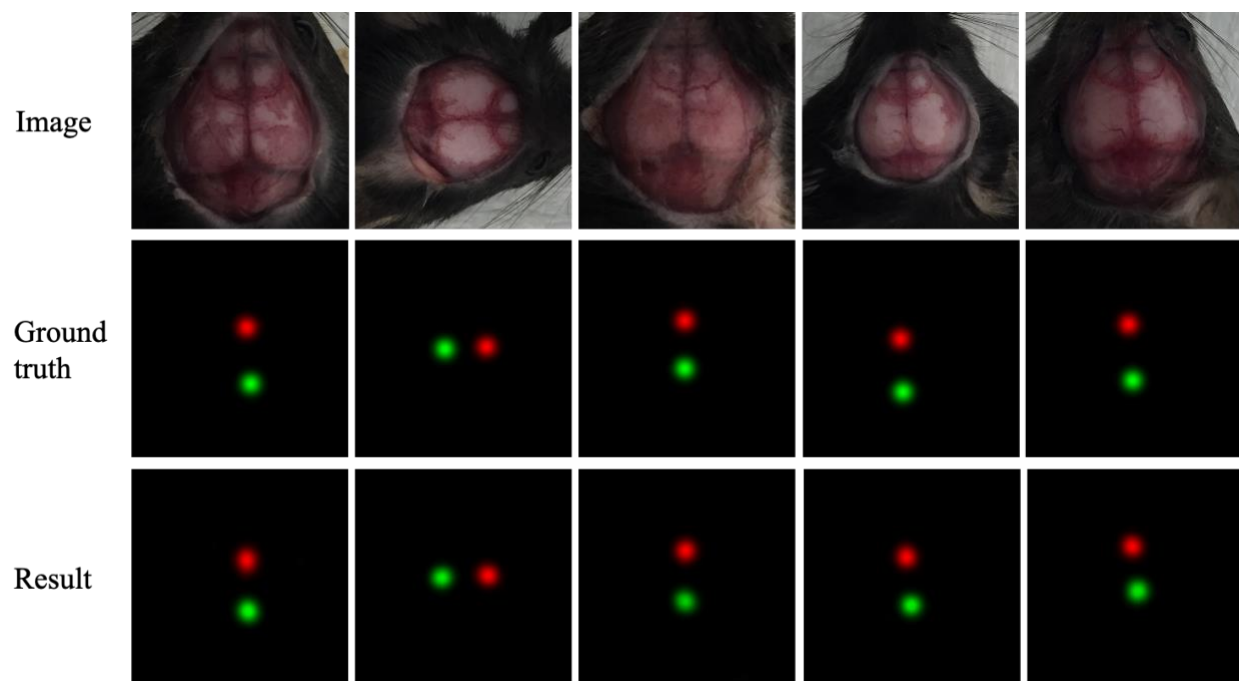

Fig 6

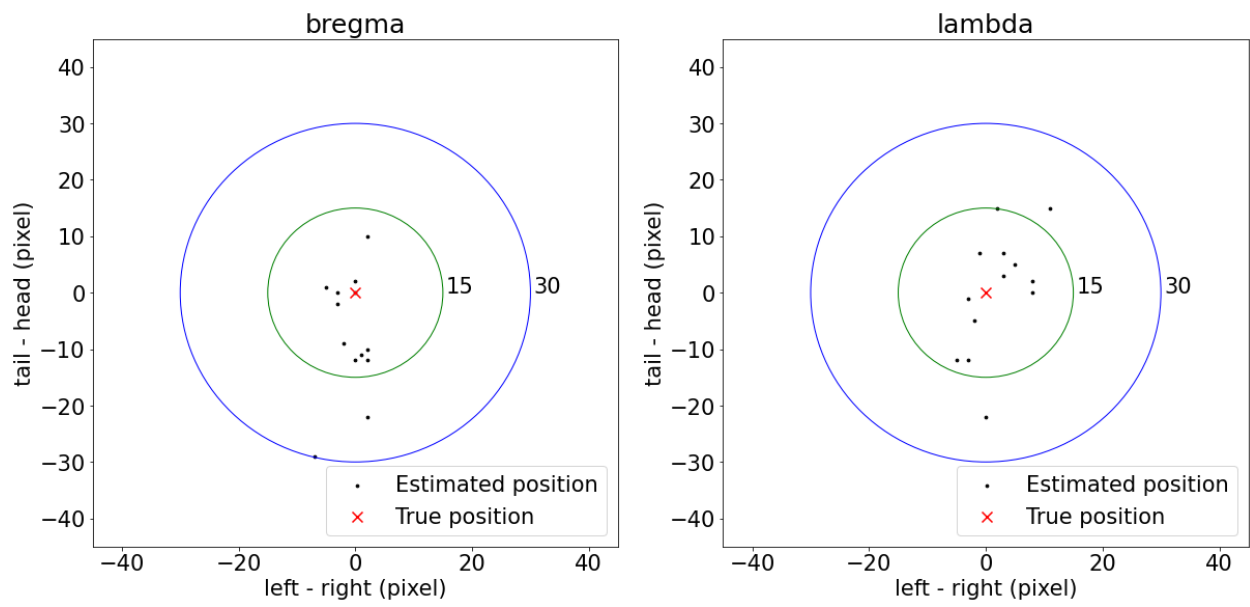

Fig 7

Supplement: S1 Raw images — (PDF) [file pone.0244378.s001.pdf]
